# Pulmonary Progressive Fibrosis in Rheumatoid Arthritis and Primary Sjogren Syndrome: Similarities and Differences

**DOI:** 10.3390/jcm13216508

**Published:** 2024-10-30

**Authors:** Andreina Manfredi, Vincenzo Venerito, Massimiliano Cazzato, Gianluca Sambataro, Umberto Zanini, Filippo Gozzi, Stefano Gentileschi, Claudia Canofari, Fabiola Atzeni, Giulia Cassone, Florenzo Iannone, Elenia Laurino, Carlo Vancheri, Fabrizio Luppi, Stefania Cerri, Marco Sebastiani

**Affiliations:** 1 Rheumatology Unit, Azienda Ospedaliero Universitaria Policlinico di Modena, 41124 Modena, Italy; 2 Department of Precision and Regenerative Medicine-Ionian Area, University of Bari, 70124 Bari, Italy; 3 Department of Clinical and Experimental Medicine, Azienda Ospedaliera Universitaria Pisana, 56100 Pisa, Italy; 4 Department of Clinical and Experimental Medicine, University of Catania, 95123 Catania, Italyvancheri@unict.it (C.V.); 5 Respiratory Diseases Unit, University of Milano-Bicocca, Fondazione IRCCS “San Gerardo dei Tintori”, 20900 Monza, Italy; 6 Respiratory Disease Unit, University of Modena and Reggio Emilia, 41125 Modena, Italy; gozzi.filippo@aou.mo.it (F.G.);; 7 Rheumatology Unit, Department of Medical Sciences, Surgery and Neurosciences, University of Siena, 53100 Siena, Italy; 8 Azienda Ospedaliera San Camillo Forlanini, Circonvallazione Gianicolense 87, 00148 Rome, Italy; 9 Rheumatology Unit, University of Messina, 98166 Messina, Italy; 10 Rheumatology Unit, Azienda Unità Sanitaria Locale di Piacenza, 29121 Piacenza, Italy; marco.sebastiani@unipr.it; 11 School of Medicine, Department of Medicine and Surgery, University of Parma, 43121 Parma, Italy

**Keywords:** interstitial lung disease, rheumatic disease, rheumatoid arthritis, primary Sjogren syndrome

## Abstract

**Background:** Progressive pulmonary fibrosis (PPF) has been associated with a worse prognosis, even when interstitial lung disease (ILD) is related to rheumatic diseases. Since many differences are detectable among rheumatic diseases in prevalence and features of ILD, we aimed to investigate features of PPF in different rheumatic diseases, namely rheumatoid arthritis (RA) and primary Sjogren’s syndrome (pSS). **Methods:** In an Italian multicentre cross-sectional study, consecutive pSS or RA patients with a diagnosis of ILD from at least two years were enrolled. For each patient, demographic, clinical, and serological data, other than chest high-resolution computed tomography and lung function tests, were recorded at the enrolment and after 2 years. **Results**: Among 232 patients, namely 156 RA-ILD and 76 pSS-ILD, a PPF was recorded in 38.8% of cases, without differences between the two diseases. Analysing patients with a PPF, usual interstitial pneumonia was significantly more frequent in RA than pSS (71.4% and 44.4%, respectively; *p* = 0.019), while ILD preceded the diagnosis of the rheumatic disease in 29.1% of RA and 89.5% of pSS (*p* < 0.001). Finally, RA patients were significantly younger than pSS at the diagnosis of the rheumatic disease (*p* < 0.001). **Conclusions:** In conclusion, although there is a similar prevalence of PPF in RA-ILD and pSS-ILD, we demonstrated for the first time that the two conditions differ in terms of radiological patterns and demographic and clinical features, suggesting that specific factors related to such diseases might influence the lung involvement over time. Prospective studies could investigate if these specificities could induce different responses to the treatment.

## 1. Introduction

Recent data suggest that an interstitial lung disease (ILD) classifiable as progressive pulmonary fibrosis (PPF) is generally associated with very poor prognosis [1], even when secondary to rheumatic diseases [1,2,3]. From a rheumatologic point of view, it is very important to underline that rheumatic diseases configure a very heterogeneous group, and these differences are well reflected also in the characterisation of lung involvement, showing specific peculiarities about both the prevalence of ILD and radiological patterns [4].

In this regard, the usual interstitial pneumonia (UIP) pattern is common in rheumatoid arthritis (RA) patients, and it is associated with severe prognosis, similar to idiopathic pulmonary fibrosis (IPF) [5,6,7]. On the contrary, in patients with connective tissue diseases, mainly systemic sclerosis (SSc), the prognosis of ILD has been reported to be independent of radiological ILD patterns [6]. Recent studies suggest that in about half of ILD cases of patients with primary Sjogren’s syndrome (pSS), a UIP is detectable, but its prognostic role needs to be confirmed yet [8,9]. In this heterogeneous context, specific features of PPF across different autoimmune rheumatic diseases may be supposed, but they have never been clearly defined.

The aim of this cross-sectional study is to investigate the clinical behaviour of lung involvement according to radiological patterns of ILD in patients with rheumatoid arthritis (RA) and pSS. Moreover, we aimed to search for possible associations between progressive fibrosing behaviour and demographic and serologic features of the disease.

## 2. Patients and Method

Consecutive patients affected by pSS or RA and classified according to the current classification criteria, namely 2010 ACR/EULAR classification criteria for RA and 2017 ACR/EULAR classification criteria for primary Sjögren’s syndrome [10,11], and with a diagnosis of ILD from at least two years were enrolled in a cross-sectional study from 10 Italian rheumatologic centres in a 12-month period (from 1 January to 31 December 2023). We used an ILD disease duration of at least 2 years according to the prescriptive criteria for nintedanib in Europe.

Lung function tests (LFTs) were periodically assessed and included % of predicted forced vital capacity (FVC) and % of predicted single-breath diffusing capacity of the lung for carbon monoxide (DLCO-SB) [12].

Demographic, clinical, and serological data, other than the more recent HRCT and LFTs, were recorded for all patients enrolled.

All participants gave their written consent, and the present study was approved by the local institutional ethics committee (approval number 108/2019).

Continuous variables were reported as median and interquartile range (IQR), and categorical variables as absolute numbers and percentages. Clinical features were reported as dichotomic or ordinal parameters. Categorical variables were analysed via the chi-square test or Fisher’s exact test when appropriate, and differences between the medians were determined using the Mann–Whitney test for unpaired samples. Then, a multivariate analysis was performed to analyse the effect of baseline features of the patient regarding the evolution to PPF [13]. Analyses were made using the Statistic for Data Analysis software (IBM SPSS statistic, version 29, Armonk, NY, USA). A *p*-value < 0.05 was considered statistically significant.

### 2.1. Radiological Evaluation

At least two chest HRCT scans for each patient were assessed by expert chest radiologists according to the Fleischner Society White Paper statement on the diagnosis of IPF [14] to confirm radiological classification. HRCT patterns of disease were recorded as definite or probable usual UIP or indeterminate for UIP. When an indeterminate UIP pattern was detected, it was further classified as nonspecific interstitial pneumonia (NSIP), fibrotic NSIP, NSIP with organising pneumonia (OP), lymphocytic interstitial pneumonia (LIP) and other patterns [14,15].

### 2.2. PPF Definition

A progressive ILD was defined in the presence of a relative decline in FVC ≥ 10% predicted and/or a relative decline in FVC ≥ 5% predicted associated with an increased extent of fibrotic changes on chest high-resolution computed tomography (HRCT) imaging in a 24 month period [3]. Changes in respiratory symptoms, namely cough and dyspnoea, were collected but not considered for the definition of PPF to reduce the possible bias due to the retrospective evaluation of symptoms. Fibrotic disease progression was confirmed at HRCT if any of the following changes were observed at follow-up, namely, an increase of traction bronchiectasis or reticular abnormalities, the appearance of new ground-glass opacity with traction bronchiectasis, new onset or increased honeycombing, and lobar volume loss.

## 3. Results

We enrolled 232 patients, 156 (67.1%) RA-ILD and 76 (32.9%) pSS-ILD, respectively (see Table 1 for details).

The two populations showed a significant difference in female/male ratio (1.48/1 and 4.1/1 in RA and pSS, respectively; *p* = 0.002) and prevalence of smokers (48.7% and 32.9% for RA and pSS, respectively; *p* = 0.03), while the median age at the enrolment in the study was similar for the two populations. In pSS, a significantly higher percentage of patients than in RA developed ILD before the diagnosis of rheumatic disease (*p* < 0.001).

Finally, respiratory symptoms and lung function, measured as FVC and DLCO, were not different between RA and pSS (see Table 1).

Among RA-ILD patients, 79/156 (50.6%) had a usual interstitial pneumonia (UIP), 32 (20.5%) an NSIP, 5 (3.2%) a combined pulmonary fibrosis with emphysema, 1 had an NSIP/OP pattern (0.6%), and 38 (28.9%) other patterns. In pSS patients, a UIP was recorded in 25/76 (32.9%) cases, an NSIP in 47 (61.8%) and an LIP in 4 (5.3%).

A fibrosing pattern was observed in 169/232 patients, respectively in 121 RA and 48 pSS individuals, respectively. Finally, 90 (38.8%) subjects had a PPF without significant differences between the two diseases (63/156 RA and 27/76 pSS patients, respectively, *p* = 0.45).

Analysing ILD progression according to HRCT patterns of ILD, patients with PPF had a UIP in 63.3% of cases, an NSIP in 30% and other patterns in 6.7%.

**Figure 1:**
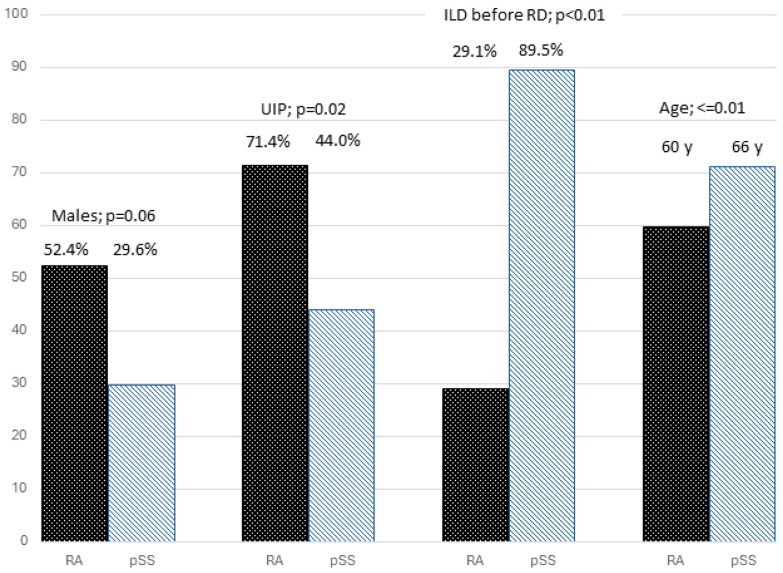
Different features of patients with RA and pSS among patients with PPF.

Moreover, we compared PPF subgroups in RA and pSS. Results are summarised in Figure 1.

Among patients with PPF, RA was characterised by a higher prevalence of UIP (*p* = 0.02). On the other hand, patients with pSS were older at the diagnosis of the rheumatic disease (RD) (*p* < 0.01), and, in a significant proportion of cases, ILD was diagnosed before the RD (*p* < 0.01).

In particular, there were no differences regarding gender and smoking habit, while in pSS patients, the diagnosis of ILD preceded that of the rheumatic disease in a higher number of patients compared to RA (*p* < 0.001), while RA patients were significantly younger at the diagnosis of the rheumatic disease (*p* < 0.001). Finally, no difference was observed regarding the age at diagnosis of ILD or regarding ILD duration.

Regarding the features of lung involvement, the UIP pattern was detected more frequently in RA than in pSS patients (*p* = 0.019). To further confirm this result, in RA patients, a PPF was observed in 57% of subjects with UIP and 23.4% without UIP (*p* < 0.001), while in pSS, a PPF was recorded in 48% of patients with UIP and 29.4% without UIP (*p* = 0.15).

**Figure 2:**
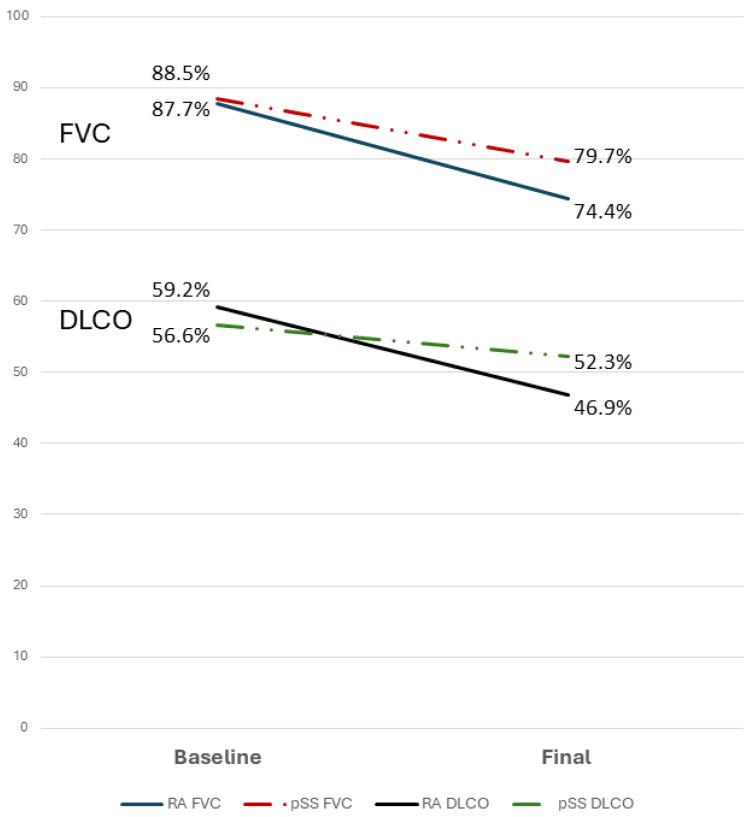
Changes in FVC and DLCO in RA and pSS in patients with PPF.

No difference was detected with regard to FVC or DLCO decline. As expected, a significant reduction of FVC during the follow-up was reported in both RA and pSS (Figure 2).

Interestingly, in non-PPF patients, FVC remained stable in RA (median decline 1%), while a significant reduction was observed in pSS (median decline −3%, *p* = 0.016). DLCO was less related to PPF behaviour; in fact, while a significant reduction was detectable in the RA-PPF group (median decline −10%; *p* < 0.001), no significant changes were observed in pSS-PPF patients (median decline −3%; *p* = 0.193).

Different distributions of decline in FVC and HRCT were detected in the RA and pSS (Table 2). The two diseases differed significantly according to progression in HRCT features, FVC% or both, *p* = 0.019.

## 4. Discussion

Interstitial lung disease (ILD) is one of the most common manifestations of systemic autoimmune rheumatic diseases [4,16,17,18,19]. However, radiological and clinical features can be highly heterogeneous, even among patients with the same rheumatic disease. These differences can become even more pronounced when patients with different systemic rheumatic disorders are compared, particularly in terms of the prevalence and progression of radiological patterns [4]. The degree of fibrotic features of ILD and the progression of the fibrosis over time at HRCT have important implications for therapeutic decisions in clinical practice. A more accurate characterisation of these differences can greatly assist in optimising patient management towards precision medicine.

In recent years, increasing attention has been given to a specific subgroup of ILDs, defined as progressive fibrosing ILD. The clinical course of these patients often resembles that of IPF, which is known to be associated with a very poor prognosis. Various definitions of progressive fibrosing ILD have been proposed, with the criteria reported in the INBUILD trial being particularly significant since they constitute the prescription criteria for the anti-fibrotic drug nintedanib in many Countries [3]. The INBUILD trial demonstrated the efficacy of nintedanib in patients with progressive fibrosing ILD other than IPF, including 170/663 patients with systemic autoimmune disease. The study enrolled individuals affected by ILD who exhibited different combinations of decline in LFTs, evaluated by FVC and/or an increase in fibrotic changes at HRCT and/or worsening in respiratory symptoms, namely cough and dyspnoea. More recently, a joint committee of many respiratory International Societies (ATS/ERS/JRS/ALAT) revised the definition with some changes, aiming to establish a globally accepted classification for these patients, proposing the term “progressive pulmonary fibrosis” [1].

These last important steps, aimed to better characterise ILDs, lead to the conclusion that in both idiopathic and secondary diseases, the overall patients’ prognosis in the presence of a PPF seems to be similar [20]. However, many points still need to be clarified among secondary PPF. Among open key questions, it could be of interest to investigate how many ILD patients develop PPF across various rheumatic diseases, which radiological patterns are most prone to progression, and the prognostic significance of changes in FVC.

Available studies show that between 30% and 40% of patients evolve in a PPF in patients with RA, pSS, and SSc, but comparative studies are lacking, and the INBUILD trial remains the only available controlled trial on PPF [9,21,22].

The aim of our study was to compare, for the first time, the features of progressive fibrosing ILD in different rheumatic diseases, namely RA and pSS, two of the most prevalent systemic autoimmune diseases potentially complicated by PPF [23,24,25,26]. Our results show that a fibrotic pattern is detected in 77.6% and 63.2% of RA and pSS patients, respectively. Notably, no significant difference was observed in the prevalence of PPF between the two diseases, with a prevalence of 40.4% and 35.5% of PPF in RA and pSS, respectively, highlighting that more than 50% of patients with a fibrosing pattern have a progression over time. Following the publication of the INBUILD study, other papers investigated the prevalence of PPF. Initial data, such as a survey of ILD experts and a US insurance claim database, suggested that the prevalence of ILDs other than IPF classified as PPF was about 18–32% [27]. In the same year, Nasser analysed data from 617 ILD patients other than IPF, finding that 27.2% of them met the INBUILD criteria [28].

In 2021, a Korean study investigated the prevalence, risk factors, and survival of PPF in non-IPF patients. The authors analysed data from 2005 to 2015, identifying 335 ILD cases secondary to autoimmune diseases, including 214 RA, 67 SSc, and 54 pSS cases. PPF-ILD was defined according to the INBUILD criteria. Among the ILDs related to autoimmune diseases, the proportions of PPF-ILD in RA, SSc, and pSS patients were 34.5%, 33.3%, and 21.7%, respectively. There was no difference in prevalence according to HRCT patterns (UIP-like: 33.1% vs. non-UIP-like: 34.9%, *p* = 0.717) [22].

The comparison of RA-ILD and pSS-ILD has led to very interesting findings: although the prevalence of PPF in the two diseases was similar, the two populations differ in terms of radiological patterns and some demographic and clinical features, suggesting the presence of specific factors related to such rheumatic diagnoses influencing the evolution of lung involvement. In RA, PPF patients are mainly characterised by a UIP pattern, whereas in pSS, the UIP pattern is less frequent than in RA, and the radiological pattern is not so relevant in the identification of patients at risk for developing a PPF. These findings highlight the importance of monitoring fibrosing non-UIP patterns in pSS patients, suggesting that these patients should receive the same attention typically given to those with a UIP pattern. The different male/female ratio in the two groups of people largely reflects the different epidemiology of the two diseases in the two sexes. On the other hand, the increased prevalence of PPF in the male gender suggested a more severe lung disease in males without allowing any other consideration of possible differences between pSS and RA. The other main difference between the two rheumatic diseases is that ILD precedes the diagnosis of the rheumatic disorder in almost 90% of pSS patients with a PPF, compared to less than 30% in RA (*p* < 0.01). It remains to be clarified if this difference was related to a specific characteristic of pSS or to a delay in diagnosis of the rheumatic disorder in patients with subclinical, lung-dominant disease. These data support the important role of the involvement of rheumatologists in the diagnostic phase, with the aim to improve the more precise classification of cases initially diagnosed as idiopathic interstitial pneumonia [29].

To our knowledge, no other studies have compared the features of different rheumatic diseases regarding PPF. Moreover, recent data on pSS could provide valuable insights. A recent Asian study aimed to compare the clinical features of PPF and non-PPF subjects using spirometry-based criteria and aimed to identify risk factors for PPF in ILD related to connective tissue diseases. The authors analysed data from 110 patients with ILD secondary to rheumatic diseases, finding that PPF was present in 27.4% of patients. The most common diagnoses were RA (33%) and pSS (30%), followed by SSc (18%) and myositis (15%). Regarding radiological patterns, the UIP pattern on chest HRCT scans was more common in the PPF group (40.7% vs. 32.5%; *p* = 0.436) [30].

In our opinion, a correct characterisation of ILD is very important for each patient affected by rheumatic disease. Certainly, an early diagnosis of ILD in rheumatic diseases should represent a task of the rheumatologist who regularly assesses the patients. However, the proper radiological and functional assessment and the early detection of patients at higher risk for progression need a multidisciplinary evaluation involving at least a rheumatologist, pulmonologist and radiologist. Equally important is that patient management involves the entire multidisciplinary team to ensure that the best therapeutic options are provided in the shortest possible time. Data from our study suggest some speculation: therapeutic approaches for RA and pSS ILD can differ according to ILD pattern. UIP patterns in RA patients suggest a combination therapy between a DMARD (usually already ongoing) and an anti-fibrotic drug. In pSS-ILD patients, a fibrotic non-UIP pattern suggests starting (or changing) an immunosuppressant, with or without an anti-fibrotic drug. Implications on the response to treatment for ILD according to the different rheumatic diseases have not been addressed by our study, but they shall be clarified only by large, specifically designed, longitudinal studies.

Our study, for the first time, suggests that the features of patients and lung involvement are different between rheumatic disorders, namely RA and pSS. Further large studies should confirm our results and evaluate possible differences with other connective tissue diseases associated with ILD, such as systemic sclerosis and inflammatory idiopathic myopathies.

## Figures and Tables

**Table 1:** Radiological, clinical and functional features of patients enrolled in the study.

	Overall Population	Rheumatoid Arthritis	Primary Sjogren’s Syndrome	*p*
Number	232	156	76	
Males, *n* (%)	78 (33.6)	63 (40.4)	15 (19.7)	0.002
Smoking habit, *n* (%)	99 (42.7)	74 (47.4)	25 (32.9)	0.037
Cough at baseline, *n* (%)	159 (68.5)	104 (66.7)	55 (72.5)	0.072
Dyspnea at baseline, *n* (%)	163 (70.2)	108 (69.2)	55 (72.5)	0.349
ILD preceding RD, *n* (%)	81 (34.9)	47 (30.1)	34 (44.7)	<0.001
UIP pattern, *n* (%)	104 (44.8)	79 (50.6)	25 (32.9)	0.012
Fibrosing, *n* (%)	169 (72.8)	121 (77.6)	48 (63.2)	0.027
Fibrosing Progressive, *n* (%)	90 (38.8)	63 (40.4)	27 (35.5)	0.566
FVC% at baseline	91 (77, 105)	91 (78, 102)	91.5 (76, 106.75)	0.881
DLCO% at baseline	58 (44, 73)	59 (47, 72)	58 (42, 75)	0.551
24 month-FVC% decline	3 (−3, 11)	3 (0, 12)	2 (−6, 10)	0.042
24 month-DLCO% decline	1 (−6, 10)	4 (−2, 13)	−1 (−14, 8)	0.003
Median age at RD diagnosis (years, IQR)	63 (53, 71)	59 (52, 67)	68.5 (59.5, 75)	<0.001
Median age at ILD diagnosis (years, IQR)	67.5 (60, 70)	68 (59, 73)	67 (62, 74.5)	0.445
Median age at enrolment (years, IQR)	70 (62, 76)	70 (62, 76)	70.5 (63, 78)	0.461

Results are reported as number (percentage) or as median (interquartile range); unless specified, data are referred to the enrolment. ILD: interstitial lung disease; UIP: usual interstitial pneumonia; RD: rheumatic disease; FVC: forced vital capacity % predicted; DLCO: diffusion lung of carbon monoxide % predicted.

**Table 2:** Decline of FVC and HRCT in pSS and RA patients.

	Decline FVC ≥ 10% Without HRCT Progression	Decline FVC ≥ 10% and HRCT Progression	Decline FVC 5–10% and HRCT Progression
RA	3 (4.8%)	38 (60.3%)	22 (33.9%)
pSS	5 (26.3%)	8 (42.1%)	6 (31.6%)

## Data Availability

The data presented in this study are available on request from the corresponding author because they are still under analysis for other studies.
